# Age, dyslexia subtype and comorbidity modulate rapid auditory processing in developmental dyslexia

**DOI:** 10.3389/fnhum.2014.00313

**Published:** 2014-05-19

**Authors:** Maria Luisa Lorusso, Chiara Cantiani, Massimo Molteni

**Affiliations:** Unit of Neuropsychology of Developmental Disorders, Department of Child Psychopathology, Scientific Institute IRCCS “E. Medea”Bosisio Parini, Italy

**Keywords:** developmental dyslexia, subgroups, rapid auditory processing, language impairment, dyslexia subtypes

## Abstract

The nature of Rapid Auditory Processing (RAP) deficits in dyslexia remains debated, together with the specificity of the problem to certain types of stimuli and/or restricted subgroups of individuals. Following the hypothesis that the heterogeneity of the dyslexic population may have led to contrasting results, the aim of the study was to define the effect of age, dyslexia subtype and comorbidity on the discrimination and reproduction of non-verbal tone sequences. Participants were 46 children aged 8–14 (26 with dyslexia, subdivided according to age, presence of a previous language delay, and type of dyslexia). Experimental tasks were a *Temporal Order Judgment* (TOJ) (manipulating tone length, ISI and sequence length), and a *Pattern Discrimination Task*. Dyslexic children showed general RAP deficits. Tone length and ISI influenced dyslexic and control children's performance in a similar way, but dyslexic children were more affected by an increase from 2 to 5 sounds. As to age, older dyslexic children's difficulty in reproducing sequences of 4 and 5 tones was similar to that of normally reading younger (but not older) children. In the analysis of subgroup profiles, the crucial variable appears to be the advantage, or lack thereof, in processing long vs. short sounds. Dyslexic children with a previous language delay obtained the lowest scores in RAP measures, but they performed worse with shorter stimuli, similar to control children, while dyslexic-only children showed no advantage for longer stimuli. As to dyslexia subtype, only surface dyslexics improved their performance with longer stimuli, while phonological dyslexics did not. Differential scores for short vs. long tones and for long vs. short ISIs predict non-word and word reading, respectively, and the former correlate with phonemic awareness. In conclusion, the relationship between non-verbal RAP, phonemic skills and reading abilities appears to be characterized by complex interactions with subgroup characteristics.

## Introduction

Developmental Dyslexia (DD) is defined as a specific disability in learning to read adequately despite at least normal intelligence, adequate instruction and socio-cultural opportunities, and the absence of sensory defects in vision and hearing (American Psychiatric Association, 1994). The prevailing views concerning the etiology of DD point to a deficit in encoding, representing and processing speech sounds (Snowling, 2001; Ramus et al., 2003; Ramus and Szenkovits, 2008). However, the question whether these difficulties reveal the core deficit of dyslexia or whether they are manifestations of a more general and basic auditory deficit is controversial.

According to Tallal's (1980) hypothesis, children with DD would be impaired in their ability to perceive auditory stimuli that have short duration and occur in rapid succession. Such a deficit at the auditory level could compromise the temporal analysis of speech at the phoneme level, and thus the building of correct phoneme representations. With such constraints, the development of language skills, both oral and written, would be difficult. Tallal and Piercy (1973a,b) revealed that children with Specific Language Impairment (SLI) have difficulties in discriminating between rapidly presented non-speech auditory stimuli, and in reproducing their order (discrimination and repetition tasks). Later, this hypothesis has been generalized to children with DD (Tallal, 1980). The procedure usually employed involves tasks that require discriminating between, or reproducing the order of, complex tones of varying frequency, manipulating both Inter-Stimulus Interval (ISI) and sound length. The difficulty in performing these tasks was interpreted by Tallal and colleagues as a deficit in extracting temporal information from short and rapid auditory stimuli, and it was referred to as deficient Temporal Auditory Processing (ATP) (Tallal, 1980). This interpretation was questioned by several researchers, who brought controversial evidence as to the exact nature of the deficit being linked to timing issues or rather to the analysis of complex stimuli (Rosen, 2003), spectral analysis and discrimination (Studdert-Kennedy and Mody, 1995), processing of stimulus streams and sluggish attentional shifting (Hari and Renvall, 2001; Lallier et al., 2012) or perceptual learning (Banai and Ahissar, 2009). As suggested by Mauk and Buonomano (2004), it is possible that in these tasks learning occurs as a result of interval-specific cognitive processes other than temporal processing *per se*; “for example, because interval discrimination tasks require comparing the test interval and a standard interval, improvement could rely on better representation of the standard interval or improved storage or retrieval from working or short-term memory” (Mauk and Buonomano, 2004, pp. 318). A prominent role of short-term memory in producing the results has also been proposed by Share et al. (2002), Tallal and Piercy (1973a,b). Heiervang et al. (2002) for instance, found that in longer trials (requiring to reproduce 3, 4, and 5 tones) children with DD made more errors compared to normally reading children. The temporal nature of the tasks has been subsequently toned down, and the hypothesis has been reworded as Rapid Auditory Processing (RAP), a definition leaving more space to different interpretations of the deficit. Despite extensive research effort, however, the specific nature of RAP problems remains ill-defined.

A first controversial point concerns the *speech-specific nature* of the deficits, as claimed by Studdert-Kennedy and Mody (1995), and Mody et al. (1997). Nowadays, the growing number of studies showing deficits concerning also the discrimination and reproduction of non-speech stimuli points toward a more general and basic auditory problem. Vandermosten et al. (2010, 2011) recently found a clear pattern in children and adults with DD, showing a temporal-specific deficit in both speech and non-speech categorization tasks. Nonetheless, the relationship between RAP deficits, phonemic awareness and reading is still a matter of debate (see Johnson et al., 2009; Malenfant et al., 2012).

A second controversy concerns the *selectivity* of the auditory processing deficit i.e., its being restricted to brief and rapidly presented stimuli. Tallal (1980) employing the Temporal Order Judgment task (TOJ) showed that children with DD performed worse than the control group in the identification of brief sounds (75 ms), but only for short Inter-Stimulus Intervals (ISIs) (8–305 vs. 428 ms). In support of the selectivity of the deficit in DD, Gaab et al. (2007) brought evidence of a disruption of cerebral regions specifically devoted to rapid auditory processing. Several results unequivocally consistent with the “restricted” RAP hypothesis have been reported, though the emphasis alternates between ISI and tone length, or both (Tallal, 1980; Reed, 1989; Heiervang et al., 2002; Cohen-Mimran and Sapir, 2007). On the other side, the findings of a second group of researchers support the hypothesis of a general auditory deficit, not restricted to short and rapid sounds (Marshall et al., 2001; Waber et al., 2001; Share et al., 2002; Bretherton and Holmes, 2003; Cantiani et al., 2010). As pointed out by Rosen (2003), group differences at long ISIs do not often emerge only because of ceiling performance. Other types of auditory processing have also been called into play, including processing of dynamic features of auditory stimuli, such as amplitude and frequency modulations (AM, FM) in the speech signal (Witton et al., 1998; Talcott et al., 2000) or sensitivity to longer time-scale patterns of intonation, rhythm and stress (Goswami et al., 2002; Pasquini et al., 2007; Thomson and Goswami, 2008; see Hämäläinen et al., 2012 for a review).

A last issue concerns the *predictive value* of measures of RAP with respect to reading and reading-related skills. Several studies found general correlations between different measures of impaired auditory processing and reading and/or phonological difficulties (Tallal, 1980; Witton et al., 1998; Ahissar et al., 2000; Marshall et al., 2001; Share et al., 2002; Hood and Conlon, 2004; Cohen-Mimran and Sapir, 2007). In many studies, however, correlations and/or predictive power were weak (Marshall et al., 2001; Share et al., 2002; Hood and Conlon, 2004) or non-significant (Reed, 1989; Heiervang et al., 2002). A recent study (Johnson et al., 2009) found that phonemic awareness predicts later RAP performance to a greater degree than the reverse. On the other hand, longitudinal studies in which behavioral and ERP responses to auditory stimuli had been recorded in newborn children with and without familial risk for language and reading disorders (Benasich and Tallal, 2002; Benasich et al., 2002; Leppänen et al., 2010) show that the infants' ability to discriminate temporal characteristics of the stimuli differs in the two groups (with and without risk) and predicts later language and reading-related skills: these findings at the very least rule out the hypothesis that RAP deficits are a consequence of reduced phonemic awareness. As a viable compromise, based on data from a longitudinal study, Boets et al. (2011) suggest a bidirectional relationship between auditory processing of non-speech stimuli and speech perception.

### How should this wide heterogeneity of results be explained?

Although the exact nature of the processes tapped by RAP tasks is a primary issue for research, the origin of the extreme variability in research findings, as described above, remains an interesting and still unanswered question. Various hypotheses have been proposed pointing to differences within the dyslexic population (McArthur and Bishop, 2001). In fact, only a subgroup of children with DD has often been found to be impaired in RAP tasks: Tallal (1980) found that only 8 (out of 20) children with DD had a clear deficit on the TOJ task. Similar within-group differences were found by Marshall et al. (2001; 4 of 17), Bretherton and Holmes (2003; 20 of 42), Ramus et al. (2003; 9 of 16), Banai and Ahissar (2004; 15 of 46), and Cohen-Mimran and Sapir (2007; 4 of 12).

First, *Age* has often been suggested to provide variability within the dyslexic group. Tallal (2000) claimed that only younger children with DD have RAP deficits, which may be explained by a maturational lag in the development of the auditory system (McArthur and Bishop, 2001; Wright and Zecker, 2004). The magnitude of this deficit is expected to diminish as children grow older: older children with dyslexia could have compensated the deficit, but only after it has compromised in a permanent way the quality of phoneme representations. Results in line with this hypothesis were found by Hautus et al. (2003), through a test of auditory temporal acuity (a gap-detection task).

Second, the *presence of language impairments* was hypothesized by several authors to be related to RAP performance (Tallal and Stark, 1982; Heath et al., 1999; Joanisse et al., 2000). In particular, Tallal and Stark (1982) did not find any tone processing deficits in reading-impaired children without concomitant oral language delay. Similarly, Heath et al. (1999) compared disabled readers with and without concomitant oral language delay in a TOJ task, and found a deficit only in the first group.

Finally, it was supposed that the RAP deficit affects only a subgroup of children with DD, based on *type of dyslexia*. Several studies suggest the existence of various subtypes of DD characterized by different cognitive and neuropsychological profiles and by different reading strategies (e.g., Bakker, 1973; Boder, 1973; Castles and Coltheart, 1993). More recently, the existence of markedly different cognitive profiles within the dyslexic population has been further confirmed (Ramus et al., 2003; Heim et al., 2008; Menghini et al., 2010). The main classification systems distinguish between dyslexic individuals with predominant difficulties in non-word reading and phonological tasks (these subtypes may be classified as L-types, phonological, dysphonetic dyslexics in Bakker's, Coltheart's and Boder's taxonomies, respectively), and dyslexic individuals who are mostly impaired in the access to the visual lexicon, as shown by their difficulties in whole-word recognition needed for reading irregular words (classified as P-types, surface or dyseidetic dyslexics). Consistent with Tallal's findings of a correlation between tone processing and non-word reading, it was assumed that only (or especially) phonological dyslexics would have a deficit in RAP (Cestnick, 2001). However, not all reports are consistent with this hypothesis: Lachmann et al. (2005) even found greater anomalies in children with dyseidetic DD compared to children with dysphonetic DD in a temporal processing task using event-related brain potentials (ERP).

Aim of the present study was to test the hypothesis that differences in age, dyslexia subtype and comorbidity with language impairments can be linked to different patterns of performance on RAP tasks. Although any subgrouping procedure may be seen as a reductive simplification of a complex, multi-factor picture (with much variability due to the specific tests and cut-offs used), our hypothesis was that a number of distinctions can highlight some crucial differences in the population. Specifically, based on previously reported findings, we expected that (a) the level of difficulty and thus of sensitivity of the different tasks would be modulated by age for children with DD in a possibly different way as compared to control children; (b) the presence of an additional language impairment would further hamper RAP performance, and (c) children with a phonological type of dyslexia would have worse RAP performance as compared to children with non-phonological dyslexia. The study is the first one, to our knowledge, to take into account all these variables in the same sample of dyslexic children. In order to avoid introducing new sources of variability in the results, only tasks that have been previously employed and well-described in the literature were used.

## Materials and methods

### Participants

Forty-six children aged between 8 and 14 years participated in the study: 26 children with DD and 20 normally reading control children. The participants in the two groups were matched for gender and age. Parental consent was obtained after the purpose and procedures of the study had been explained. The study had been approved by the Ethics Committee of the Institute according to standards of the Helsinki Declaration (1964).

Children with DD included in the sample had been referred to the Unit of Cognitive Psychology and Neuropsychology of the institute because of learning difficulties. All children had been diagnosed as dyslexic based on standard inclusion and exclusion criteria (ICD-10; World Health Organization, 1992). Their performance in reading was two (or more) standard deviations below the mean in at least one of the age-standardized Italian reading tests included in the battery (word, non-word and text reading), and their non-verbal or performance IQ was above 85. Performance IQ was estimated by the Italian adaptation of the Wechsler Intelligence Scale for Children-revised (WISC-R; Wechsler, 1994) (*n* = 15), or Cattell's “Culture Free” test (Cattell, 1979) (*n* = 11). All children attended mainstream schools (as is usual in the Italian educational system), and none of them had started remediation programs at the time of participation in the study. Comorbidity with ADHD or other psychopathological conditions was excluded, based on standard diagnostic criteria (DSM-IV; American Psychiatric Association, 1994).

Control children were recruited in local schools. They all performed normally in a text reading task, and their performance IQ (Cattell's “Culture Free” test) was above 85. Participants' characteristics compared with unpaired *t*-tests are shown in Table 1. No group difference emerged in age, but there was a significant difference in performance IQ.

**Table 1:** **Participant characteristics (*p*-values indicating significant group differences are marked in bold)**.

**Group**	**DD (*N* = 26) Mean (*SD*)**	**Control (*N* = 20) mean (*SD*)**	**Group comparison *F(df), p***
Male	21	15	
Female	5	5	
Age in months	128.58 (23.04)	133.20 (22.27)	0.468 (1.45), 0.497
Performance IQ^a^	102.96 (8.29)	112.00 (11.36)	9.737 (1.45), **0.003**
READING: accuracy^b^	−2.94 (1.68)	0.09 (0.57)	59.604 (1.45), **< 0.001**
READING: speed^b^	−1.89 (1.74)	0.31 (0.29)	31.241 (1.45), **< 0.001**

a Scores at WISC-R or Cattell's “Culture Free” test;

b Scores are expressed as Z-scores in the text reading task.

#### Subgroups

Within the dyslexic sample, two subgroups based on the presence or absence of a previous language delay were created, after an accurate analysis of clinical records: all children had been diagnosed at the Institute following the same diagnostic protocols; thus, a detailed anamnestic record was available including in-depth information about language development. Inclusion criteria were previous diagnoses of Language Impairment (LI) (a diagnosis of LI is made when at least two scores on a standardized battery of receptive and expressive language are below 2 SDs with respect to age norms) and/or reports of significant delays (reported delays were considered significant if the main linguistic milestones were acquired with at least one year delay with respect to normal development) in early vocabulary and syntactic development, in addition or not to a history of speech and language therapy. Transient phonetic/articulatory difficulties without any additional linguistic problem were not considered sufficient for inclusion in the DD-LI group, even if speech therapy had been delivered. Based on these elements, it was ascertained that 10 children had had a clear previous language impairment (LI) and 11 children had never presented linguistic difficulties (noLI). For five of the children, available information was not sufficient to decide on the presence of a previous linguistic impairment, so they were not classified and not included in the analysis. Hearing tests had been performed for all children with a former diagnosis of SLI as part of the diagnostic procedure. For dyslexic participants, hearing tests were performed anytime there was a reason to suspect that a hearing problem may be present (based on parents' reports or on the clinicians' assessment). Only children for whom no report of a hearing loss was recorded were included in the study.

Further, two subgroups based on type of dyslexia were created. This division was based on the difference in accuracy (z-scores) between word and non-word reading (with at least 0.5 difference in z-scores)^1^. This procedure is similar, although not identical, to the regression procedure suggested by Castles and Coltheart (1993) and followed by Ziegler et al. (2008) and Peterson et al. (2013), to select “relative phonological” and “relative surface” dyslexics. Children with “phonological DD” performed worse when reading non-words, while children with “surface DD” performed worse when reading words. Accordingly, a total of 10 children were assigned to the subgroup of phonological DD, 12 were assigned to the subgroup of surface DD and 4 could not be classified.

Table 2 shows the combinations of z-scores expressing accuracy and speed in reading words and non-words, for each participant. It can be easily seen that the great majority of children had difficulties with both kinds of stimuli, and that a “relative” rather than a “pure” subtype classification is the best choice. As to accuracy vs. speed scores as the basis for classification, it can be seen that both variables would allow to identify (largely but not completely overlapping) subgroups with similar numbers of participants. It was decided to use accuracy rather than speed scores based on previous studies in which a subdivision according to accuracy scores highlighted strong and reliable differences in visual and auditory attention (Facoetti et al., 2010; Franceschini et al., 2012), i.e., in low-level processing skills.

**Table 2:** **z-scores for word and nonword reading for each participant (DD group)**.

				**Word reading accuracy**	**Word reading speed**	**Nonword reading accuracy**	**Nonword reading speed**
Subtype	Surface	1		−4.78	−3.50	−3.86	−1.83
		2		−6.21	−4.73	−4.69	−2.45
		3		−4.88	−1.48	−3.51	−2.33
		4		−6.90	−1.81	−4.97	−0.22
		5		−8.71	−8.18	−3.53	−5.77
		6		−4.50	−4.20	−2.17	−2.14
		7		−3.50	−1.02	−0.68	−0.34
		8		−5.20	−5.60	−3.60	−3.36
		9		−4.86	−1.40	−2.21	−0.85
		10		−3.11	−1.45	−0.48	−1.14
		11		−1.44	−3.39	−0.82	−2.48
		12		−2.60	−4.70	−0.40	−5.00
		Total	*N*	12	12	12	12
			Mean	−4.7242	−3.4550	−2.5767	−2.3258
	Phonological	1		−2.64	−1.05	−4.97	−0.62
		2		−3.66	−1.40	−4.28	−2.06
		3		−0.92	−1.80	−2.71	−1.04
		4		−0.14	−6.63	−2.47	−5.40
		5		−0.69	−2.14	−1.28	−0.52
		6		−1.68	−2.59	−2.92	−4.89
		7		−2.18	−5.76	−3.36	−7.50
		8		−1.88	0.09	−3.36	0.50
		9		0.24	−0.43	−4.07	−0.18
		10		−0.86	0.44	−5.81	0.63
		Total	*N*	10	10	10	10
			Mean	−1.4410	−2.1270	−3.5230	−2.1080
	Total	*N*		22	22	22	22
		Mean		−3.2318	−2.8514	−3.0068	−2.2268

Subgroup characteristics and one-way ANOVA comparisons are shown in Table 3. Subgroup comparisons reflected the inclusion criteria in each subgroup (see comparison for non-word reading accuracy in the type-of-dyslexia subgroups). All subgroups resulted comparable for IQ. Generally, children with DD-noLI had lower performances in reading and reading-related tasks than children with DD+LI (these differences reached significance for reading accuracy and short-term memory scores). No significant differences emerged in overall reading and reading-related tasks when comparing subgroups based on type of dyslexia (except for the difference in word reading accuracy scores). However, an interesting pattern emerged for the phonemic awareness scores, that was further explored. A repeated-measure ANOVA was performed, entering type of phonemic awareness task (see following section for a description) as within-subject factor (phoneme deletion vs. phonemic blending) and type of dyslexia as between-subject factor. A significant interaction between type of phonemic awareness task and type of dyslexia was found, *F*_(1, 20)_ = 5.36, *p* < 0.05; ŋ^2^_*p*_ = 0.211, suggesting different phonemic awareness difficulties in the two subgroups. Namely, children with phonological dyslexia had similar performances in the two phonemic awareness tasks, whereas children with surface dyslexia were more impaired in phonemic blending than in phoneme deletion [*F*_(1, 11)_ = 21.47, *p* = 0.001; ŋ^2^_*p*_ = 0.661].

**Table 3:** **Subgroup characteristics (*p*-values indicating significant group differences are marked in bold)**.

**Grouping Criteria**				**Comparison between dyslexic subgroups**
Presence/absence of previous language impairment		**DD+LI**	**DD-no LI**	***F(df), p***
	*N*	10	11	
	AGE^a^	130.2 (23.90)	122.6 (20.19)	0.62 (1.20), 0.442
	*IQ*^b^	107.2 (7.24)	101.0 (8.73)	3.01 (1.20), 0.099
	READING: accuracy^c^	−2.14 (0.69)	−3.67 (1.42)	9.53 (1.20), **0.006**
	READING: speed^c^	−1.74 (1.61)	−2.72 (2.08)	1.44 (1.20), 0.244
	SHORT−TERM MEMORY^d^	−0.48 (0.62)	−1.078 (0.45)	6.01 (1.19), **0.025**
	PHONEME DELETION^e^	1.50 (1.43)	3.27 (2.49)	3.88 (1.20), 0.064
	PHONEMIC BLENDING^e^	3.50 (2.63)	5.36 (3.44)	1.91 (1.20), 0.183
Type of dyslexia		**“Phonological” DD**	**“Surface” DD**	***F(df), p***
	*N*	10	12	
	AGE^a^	128.3 (17.88)	133.2 (27.71)	0.236 (1.21), 0.632
	IQ^b^	102.3 (9.02)	102.2 (8.07)	0.00 (1.21), 0.989
	READING: accuracy^c^	−2.66 (0.85)	−3.51 (1.50)	2.505 (1.21), 0.129
	READING: speed^c^	−1.92 (2.32)	−2.76 (1.43)	1.075 (1.21), 0.312
	NON−WORD READING: accuracy^f^	−3.52 (1.30)	−2.57 (1.67)	2.115 (1.21), 0.161
	WORD READING: accuracy^f^	−1.44 (1.19)	−4.72 (1.96)	21.225 (1.21), **< 0.001**
	SHORT−TERM MEMORY^d^	−1.03 (0.51)	−0.91 (0.39)	0.331 (1.20), 0.572
	PHONEME DELETION^e^	3.00 (2.45)	1.67 (1.87)	2.09 (1.21), 0.163
	PHONEMIC BLENDING^e^	4.00 (2.87)	5.33 (3.23)	1.03 (1.21), 0.323

a Age in months;

b Scores on WISC-R or Cattell's “Culture Free” test:

c Global scores were created considering both scores on word and non-word reading and on text reading. Mean scores expressed as Z-scores were calculated separately for accuracy and speed;

d Z-scores;

e raw scores (number of errors);

f Z-scores in the word and non-word reading tasks.

The distribution of dyslexia subtypes in the two groups with/without previous language delay was not significantly different, χ^2^(1, *N* = 19) = 1.269, *p* > 0.05.

### Tasks

#### Reading and reading-related tasks

*Reading skills* were assessed through two different tasks:

- “Prove di lettura MT per la scuola elementare-2” (Reading tests for primary school, Cornoldi et al., 1998) and “Nuove prove di lettura MT per la scuola media inferiore” (New reading tests for secondary school, Cornoldi and Colpo, 1995), widely used Italian tests providing accuracy and speed scores in reading aloud age-normed texts.
- “Batteria per la valutazione della dislessia e disortografia evolutiva” (Battery for the assessment of Developmental reading and spelling disorders, Sartori et al., 1995). In particular, speed and accuracy z-scores were computed for single word (4 lists of 28 words) and non-word reading (3 lists of 16 non-words).

Two *phonemic awareness* tests were taken from an unpublished battery (Cossu et al., 1988):

- “Phoneme deletion”: canceling the first two phonemes of orally given words
- “Phonemic blending”: integrating sequentially presented phonemes into words

Scores for each test are expressed as number of errors on 20 words. Only age means and cut-off scores are provided as normative data.

*Short-term memory* was assessed by a digit span subtest, comprising Digits Forwards and Digits Backwards. For the children who had undergone intelligence testing with the WISC-R, the weighted score of the Digit Span subtest was recorded. For the other participants, digit span was assessed by a subtest of TEMA (Test di Memoria e Apprendimento, an Italian adaptation of TOMAL, Reynolds and Bigler, 1994), and recorded as z-scores.

#### Experimental tasks

The experimental tasks were created based on well-established protocols described in the literature. Moreover, they had already been used in a previous study by the authors (Cantiani et al., 2010). Only nonspeech processing skills were addressed, in order to avoid direct influences from linguistic or phonological deficits. All stimuli were digitally generated using Praat software (www.praat.org) and were presented to each child on an ASUS computer by means of E-prime Experiment Generator and Controller software (Schneider et al., 2002).

The *Temporal Order Judgment (TOJ) task* (Tallal and Piercy, 1973a,b; Tallal, 1980) was constructed using two complex tones composed of frequencies within the speech range. The two tones differed in the fundamental frequency (Fo = 100 Hz for the low tone and Fo = 305 Hz for the high one), and tone duration for both tones was either 75 or 250 ms. Children were instructed to indicate the order of the tones after each trial by pressing a yellow key for the “low” tone and a blue key for to the “high” tone. The same experimental paradigm was used in two different tasks: a Rapid Temporal Order Judgment (Rapid-TOJ) task, in which ISI was manipulated, and a Temporal Order Judgment Memory (TOJ-Memory) task, in which the number of elements to keep in memory was manipulated.

In the *Rapid Temporal Order Judgment (Rapid-TOJ) task*, stimulus pairs were created by pairing the two stimuli in all four possible combinations (AA, AB, BB, BA) with different insterstimulus intervals: 8, 15, 30; 60, 150, 305, and 428 ms, and presented randomly. A short training with visual and verbal feedback was given to familiarize the children with the task. First, each tone was demonstrated separately seven times, and participants had to answer by pressing the corresponding key. Then, single tones were presented in random order. This training was continued for a maximum of 48 trials or until a criterion of 20 correct responses in a series of 24 consecutive stimuli was reached (*p* < 0.001 Binomial Test). In the last phase of training, participants were trained to respond to each of the four possible stimulus patterns by pressing the keys in the correct order. There were four demonstrations by the experimenter, followed by eight trials in which participants responded independently. An inter-stimulus interval (ISI) of 428 ms was employed during training. After the training session, 24 similar trials were given without feedbacks. Children were then tested on two-element stimulus patterns with ISIs of 8, 15, 30, 60, 150, and 305 ms. Each subject received a total of 24 two-element patterns, four for each ISI, with random presentation order. This training and testing procedure was carried out twice, once for each of the two stimulus durations: 75 and 250 ms (presentation order for the two blocks was balanced across participants).

In the *Temporal Order Judgment Memory (TOJ-Memory) task*, stimulus sequences consisted of four and five elements created as random combinations of the two complex tones, with a fixed ISI of 428 ms. Two different blocks were presented in a counterbalanced order: one included 10 four-tone sequences and one included 10 five-tone sequences. Both blocks were preceded by training including one trial demonstrated by the experimenter, and three trials in which children responded independently and feedback was given. The whole training and testing procedure was carried out twice, once for each of the two stimulus durations: 75 and 250 ms (order counterbalanced across participants). Data from 2-stimulus series with 428 ms ISI from the Rapid-TOJ task were included in the analyses of the TOJ-Memory task, so as to increase the range of sequence lengths and analyze memory effects on performance (stimulus sequences of two, four and five elements).

In the *Pattern Discrimination Task* (adapted from Kujala et al., 2000) a simple behavioral procedure was adopted, requiring the children to discriminate four-tone rhythmic patterns. The stimulus patterns consisted of four synthetically generated tones (500 Hz in frequency and 30 ms in duration) separated by different ISIs (50 ms; 150 ms; 200 ms). Two different stimulus patterns (rhythms) were created by changing the order of the ISIs, and separately recorded on audio files:

- Rhythm A: sound—200 ms ISI—sound—50 ms ISI—sound—150 ms ISI—sound
- Rhythm B: sound—200 ms ISI—sound—150 ms ISI— sound— 50 ms ISI—sound

The two rhythms were paired in all four possible combinations (AA, AB, BB, BA) with 700-ms intervals. The children listened to the pairs of rhythms and were requested to say whether the two rhythms were equal (50%) or different (50%). The answers were recorded by the experimenter by pressing different keys on the computer keyboard. During the testing phase a fixation point was shown on the computer screen. The task, composed of 24 trials, was preceded by two different training phases: a passive training including 8 trials (two for each combination) demonstrated by the experimenter, and an active training including 4 trials (one for each combination), in which participants responded independently and feedback was given.

#### Apparatus and procedures

All testing was conducted individually in a quiet room. Experimental and reading tasks were presented in a single session, with a total duration of about 1 h and a half. Task sequence was counterbalanced within participants to control for fatigue effects. The stimuli of the RAP tasks were presented binaurally through headphones (Sennheiser HD270) with an intensity of approximately 60 dB. All the responses were recorded via the computer keyboard.

## Results

### Data analyses

In the first part of this section, the results of the whole sample of dyslexic participants on the three tasks are compared with those of control participants. Two separate ANOVAs (repeated measures GLM) were performed for the two different TOJ tasks (Rapid-TOJ, TOJ-Memory), and a univariate ANOVA was performed for the Pattern Discrimination task, considering the mean percentage of correct answers. In all ANOVAs, Group (dyslexic vs. control participants) was entered as between-subject factor and Age as a covariate, while within-subject factors differed according to the specific task. For the Rapid-TOJ task, accuracy on trials with short ISIs (from 8 to 30 ms inclusive) were compared with accuracy on trials with longer ISIs (60–428 ms inclusive). The cut-off was set at 40 ms as this time frame was suggested to be crucial for speech discrimination (Fitch et al., 1997). This subdivision was similar to that used in previous studies (e.g., Heath et al., 1999; Cohen-Mimran and Sapir, 2007). The adequacy of this 40 ms cut-off was empirically confirmed by a preliminary analysis on the single ISI values. Due to the significant difference in performance IQ between groups (control children had higher IQs), Pearson's bivariate correlations were first computed between performance-IQ measures and all experimental variables. Since significant correlations (*p_s_* < 0.05) were found for the TOJ-Memory task, IQ was entered as a covariate for this task. The “Delaney-Maxwell” method was applied to both IQ-scores and Age, in order to center the mean of the covariates, thus avoiding distortions of the main effects (Delaney and Maxwell, 1981). Specifically, the measure used as a covariate was the deviation of each individual score with respect to the mean score in the whole sample.

The second part of this section will focus on the dyslexic group, subdivided according to the presence/absence of a previous language impairment, and to type of dyslexia. Again, separate ANOVAs were performed for the three tasks, considering the mean percentage of correct answers. For each task, two different ANOVAs were performed, first with Language (presence vs. absence of language delay) and then with Type of Dyslexia (surface vs. phonological dyslexia) as between-subject factors. The results of control participants will be shown in the graphs as a reference point, but will not be included in the analyses. Due to the high correlations between the three tasks (all *p_s_* < 0.001), no statistical corrections were employed to adjust for multiple analyses. One-tailed *p*-values are reported (as specified in the text) when clearly unidirectional hypotheses were considered. Two-tailed *p*-values are to be intended when not otherwise specified. Due to the limited number of participants in each subgroup, all analyses showing significant differences were repeated with nonparametric statistics, and only the results that were confirmed by nonparametric tests are reported here.

Finally, correlations between measures of RAP variables and reading, phonemic awareness and short-term memory scores will be illustrated, both concerning the whole sample and the dyslexic group.

### Comparing children with/without dyslexia

#### Rapid-TOJ task

**Figure 1:**
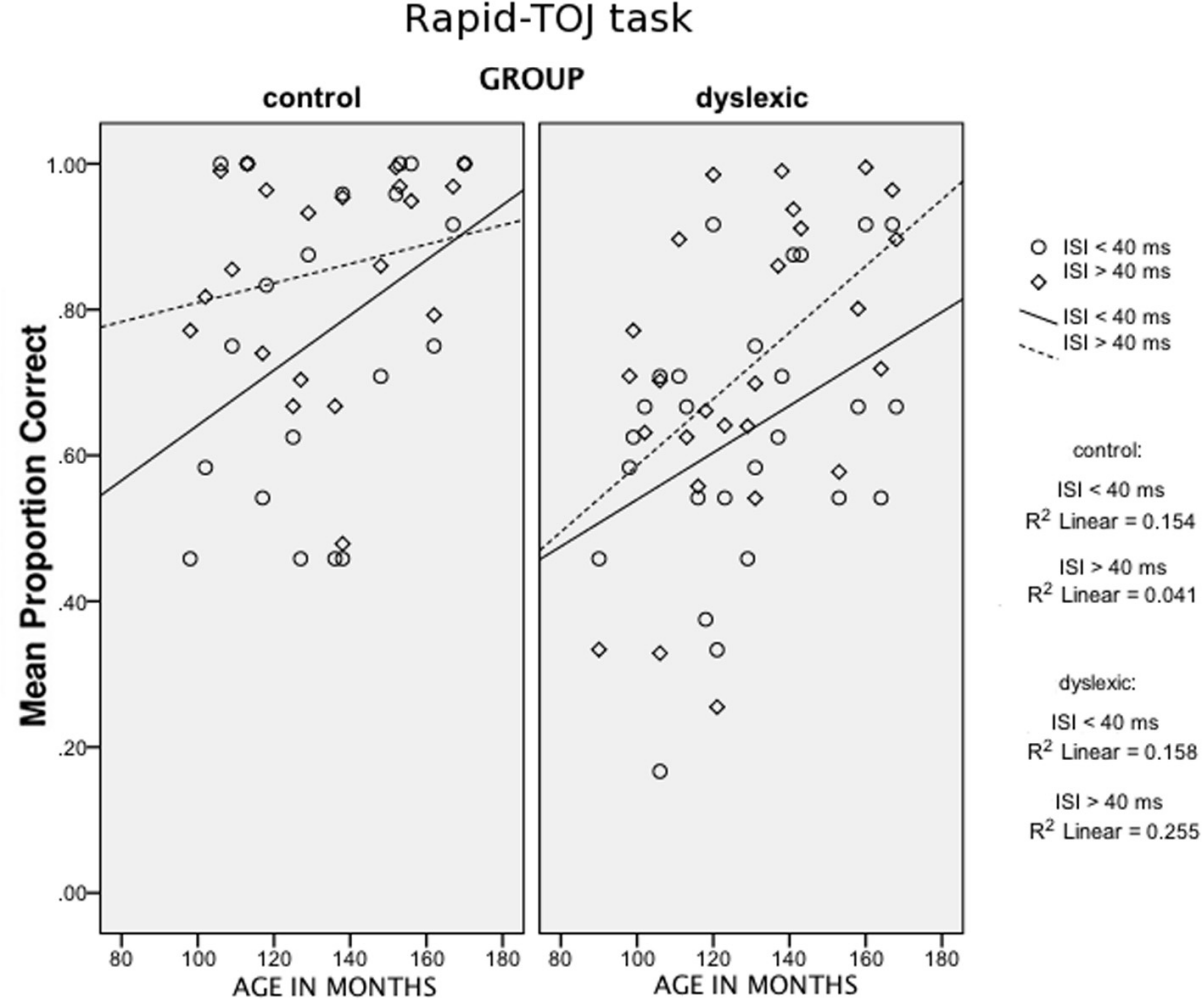
**Scatter plots of Age (in months, x-axis) against the mean proportion of correct answers in the Rapid-TOJ task (y-axis), for short and long-ISI conditions, for the two groups (control children and children with DD)**.

In addition to the described between-subject factor Group and the covariate Age, two within-subject factors were considered: Stimulus Length (75 vs. 250 ms) and Interstimulus Interval (ISI) (short ISIs: 8–30 ms vs. long ISIs: 60–428 ms). The main effect of Group reached statistical significance, *F*_(1, 42)_ = 6.13, *p* < 0.01 [1-tailed]; ŋ^2^_*p*_ = 0.127, with fewer correct responses for the dyslexic group compared to the control group. Significant effects were found for Stimulus Length, *F*_(1, 42)_ = 18.80, *p* < 0.001; ŋ^2^_*p*_ = 0.309, and ISI, *F*_(1, 42)_ = 29.77, *p* < 0.001; ŋ^2^_*p*_ = 0.415. A close-to-significance interaction (Stimulus Length × ISI: *F*_(1, 42)_ = 3.97, *p* = 0.053; ŋ^2^_*p*_ = 0.086) indicated a general greater difficulty associated with the processing of short and rapid sounds. Finally, an interaction ISI × Group × Age emerged, *F* = 6, 70, *p* < 0.05, ŋ^2^_*p*_ = 0.138. This interaction is illustrated in Figure 1. No further interactions were found of any variable with either Group or Age (all *p_s_* > 0.05).

#### TOJ-memory task

**Figure 2:**
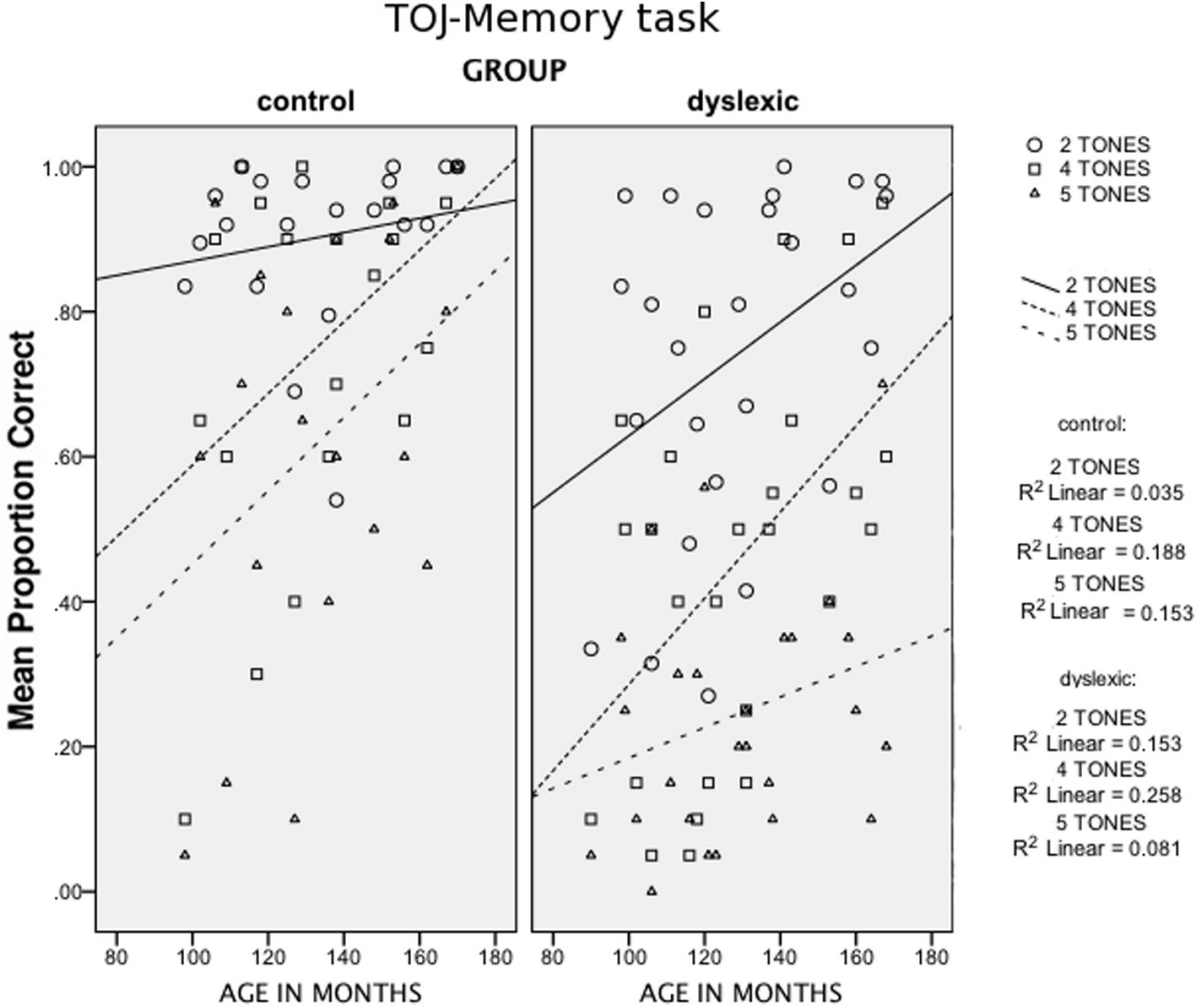
**Scatter plots of Age (in months, x-axis) against the mean proportion of correct answers in the TOJ-Memory task (y-axis), for the different Sequence Length conditions (2, 4, and 5-tone conditions) for the two groups (control children and children with DD)**.

In addition to Group and Age, two within-subject factors were considered: Stimulus Length (75 vs. 250 ms) and Sequence Length (2 vs. 4 vs. 5 elements). A significant main effect emerged for Group, *F*_(1, 41)_ = 14.93, *p* < 0.001 [1-tailed]; ŋ^2^_*p*_ = 0.267. Moreover, a significant interaction between Group and Sequence Length was found, *F*_(2, 82)_ = 5.53, *p* < 0.005 [1-tailed]; ŋ^2^_*p*_ = 0.119, due to a worse drop in performance from the 2-tone-sequences to the 5-tone-sequences for children with DD as compared to control children. A further interaction with Age [Group × Age × Sequence Length, *F*_(2, 82)_ = 2.78, *p* < 0.05 [1-tailed]; ŋ^2^_*p*_ = 0.064), indicates different performance patterns within the dyslexic group, as shown in Figure 2.

#### Pattern discrimination task

Similarly to the results obtained in the TOJ tasks, the main effects of Group, *F*_(1, 45)_ = 28.42, *p* < 0.001 [1-tailed]; ŋ^2^_*p*_ = 0.404, and Age, *F*_(1, 45)_ = 5.25, *p* < 0.05, ŋ^2^_*p*_ = 0.111, but not the interaction between Group and Age, reached statistical significance.

### Comparing subgroups of children with DD

**Figure 3:**
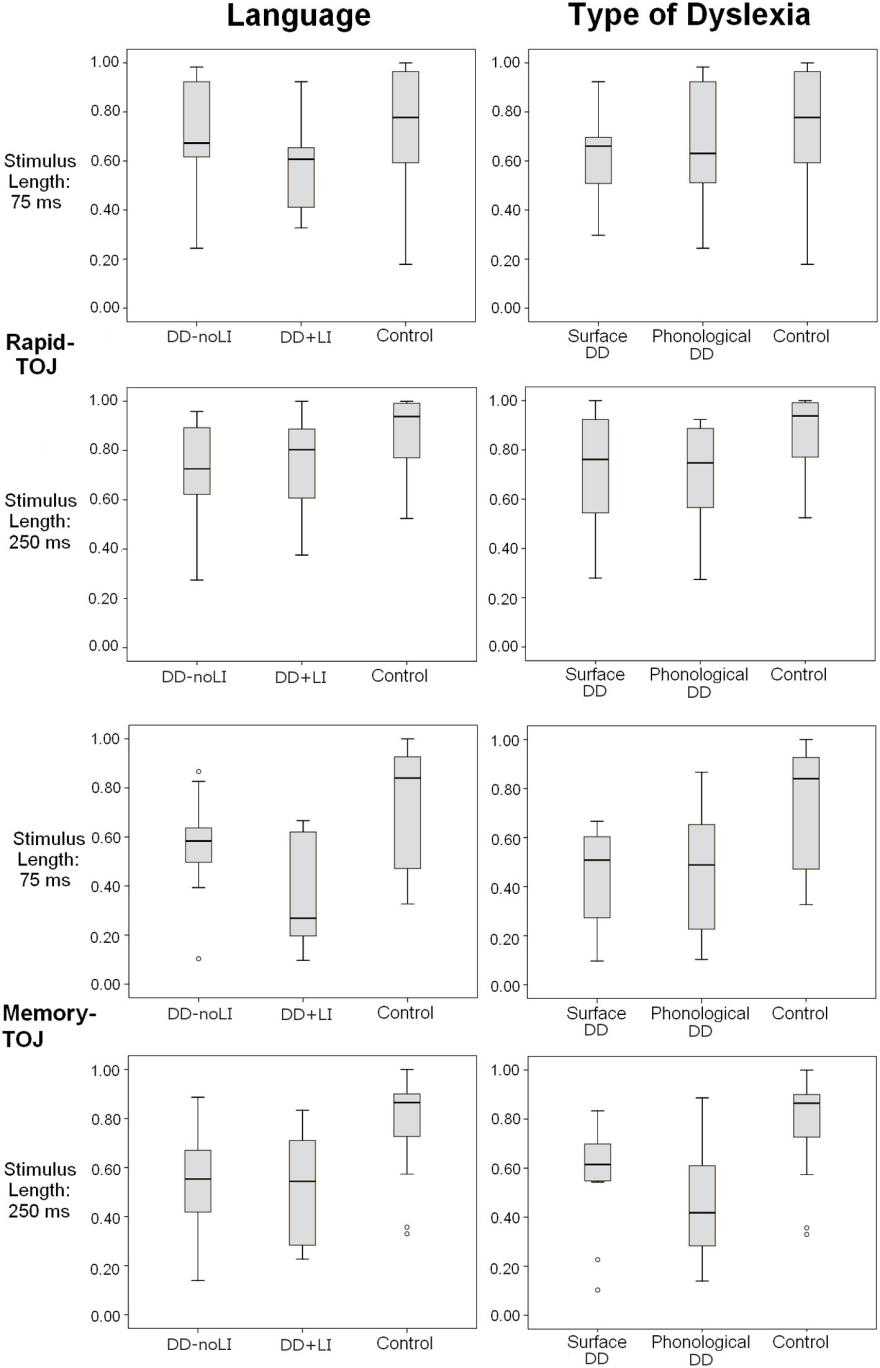
**Box plots displaying the distribution of individual scores in different subgroups (based on Language and Type of Dyslexia) for the Rapid-TOJ and the Memory-TOJ tasks**. Empty circles correspond to outliers.

No main effects of the subgroup divisions (Language and Type of Dyslexia) emerged in any task (all *p_s_* > 0.2). However, significant interactions were found, that will be presented separately for the Rapid-TOJ task and the TOJ-Memory task. Figure 3 shows the distribution of the main variables in the subgroups, and outliers for each group.

#### Rapid-TOJ task

**Figure 4:**
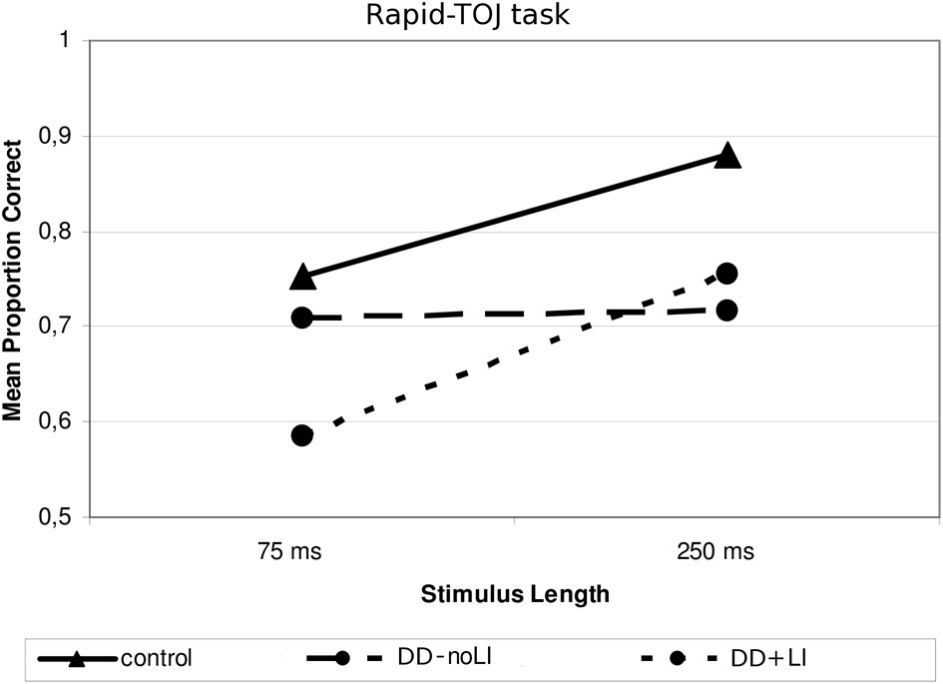
**Mean proportion of correct answers in the Rapid—TOJ task for the subgroups divided according to Language**.

Concerning the subdivision *Presence/absence of a previous language delay*, a significant interaction emerged between Language and Stimulus Length, *F*_(1, 19)_ = 4.85, *p* < 0.05; ŋ^2^_*p*_ = 0.204. As Figure 4 shows, children with DD+LI, similarly to control children, performed worse when sounds were shorter (*M* = 0.585; *SD* = 0.188) and better when sounds were longer (*M* = 0.755; *SD* = 0.208), *F*_(1, 9)_ = 7.09, *p* < 0.05; ŋ^2^_*p*_ = 0.441, while children with DD-noLI had similar performances in the two conditions (shorter sounds: *M* = 0.708; *SD* = 0.226; longer sounds: *M* = 0.717; *SD* = 0.213), *F*_(1, 10)_ = 0.062, *p* > 0.05. As compared to controls, a difference approaching statistical significance was found for children with DD+LI in the 75-ms-tone condition, *F*_(1, 30)_ = 3.919, *p* = 0.058, ŋ^2^_*p*_ = 0.123 while a significant difference for children with DD-noLI was found only in the long-tone condition, *F*_(1, 31)_ = 6.803, *p* < 0.05 (ŋ^2^_*p*_ = 0.190). No other significant interactions emerged. Concerning the subdivision *Type of dyslexia*, no significant interactions were found (all *p_s_* > 0.1).

#### TOJ-memory task

**Figure 5:**
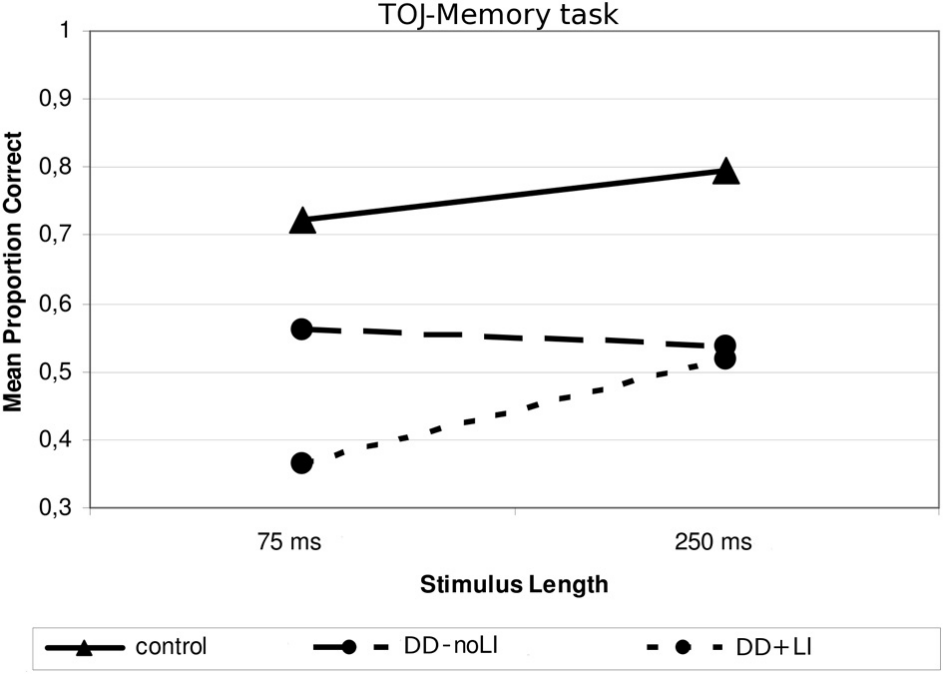
**Mean proportion of correct answers in the TOJ—Memory task for the subgroups divided according to Language**.

Concerning the subdivision *Presence/absence of a previous language delay*, a significant interaction between Language and Stimulus Length was found, *F*_(1, 19)_ = 9.00, *p* < 0.01; ŋ^2^_*p*_ = 0.321. As Figure 5 shows, children with DD+LI, comparably to control children, performed worse when sounds were shorter (*M* = 0.365; *SD* = 0.218) and better when sounds were longer (*M* = 0.517; *SD* = 0.218), *F*_(1, 9)_ = 9.58, *p* < 0.05 (ŋ^2^_*p*_ = 0.516), while children with DD-noLI had similar performances in the two conditions (shorter sounds: *M* = 0.562; *SD* = 0.206; longer sounds: *M* = 0.538; *SD* = 0.208), *F*_(1, 10)_ = 0.51; *p* > 0.05. Compared to the control children, significant differences were found in both conditions for children with DD+LI [75-ms-tone condition, *F*_(1, 30)_ = 15.239, *p* = 0.001, ŋ^2^_*p*_ = 0.352; 250-ms-tone condition, *F*_(1, 30)_ = 12.90, *p* = 0.001, ŋ^2^_*p*_ = 0.315), while a significant difference for children with DD-noLI was found only in the long-tone condition, *F*_(1, 31)_ = 12.15, *p* < 0.01, ŋ^2^_*p*_ = 0.295. No other significant interactions emerged. As shown in Figure 3, two outliers may be identified in the DD-noLI subgroup processing short sounds. Yet, after excluding these subjects in the main ANOVA, the interaction Language × Stimulus Length remains significant, *F*_(1, 17)_ = 8.45, *p* = 0.01; ŋ^2^_*p*_ = 0.332.

**Figure 6:**
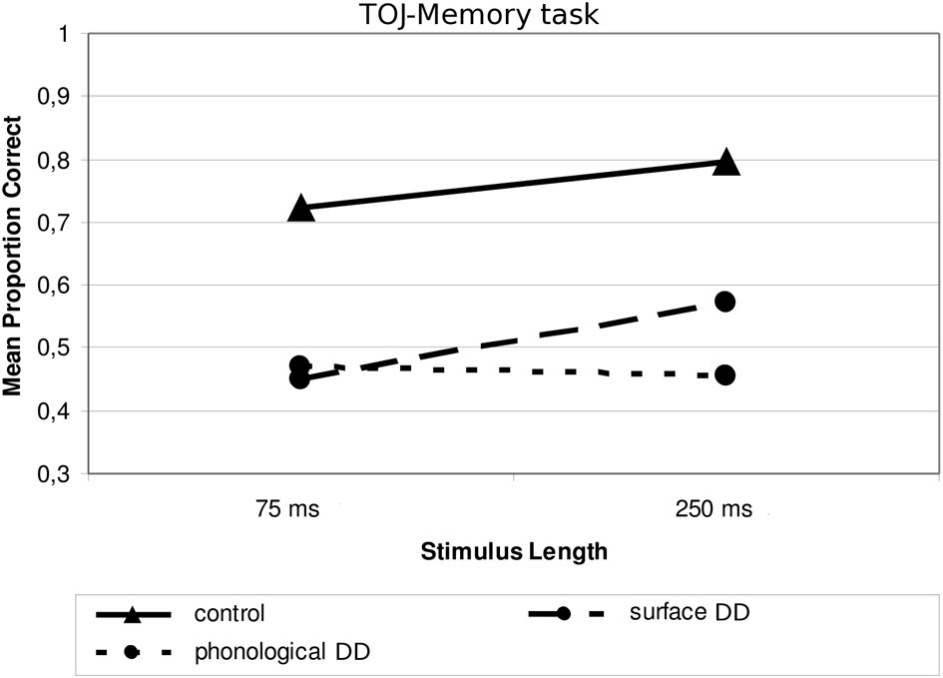
**Mean proportion of correct answers in the TOJ—Memory task for the subgroups divided according to Type of Dyslexia**.

Concerning the subdivision *Type of dyslexia*, the only significant interaction concerned Type of Dyslexia and Stimulus Length, *F*_(1, 20)_ = 4.49, *p* < 0.05; η^2^_*p*_ = 0.184. In this case, children with surface DD (similarly to children with DD+LI and to controls) performed worse with shorter (*M* = 0.449; *SD* = 0.193) than with longer tones [*M* = 0.572; *SD* = 0.210, *F*_(1, 11)_ = 7.041, *p* < 0.05, ŋ^2^_*p*_ = 0.390], while children with phonological DD performed similarly in the two conditions (shorter sounds: *M* = 0.470; *SD* = 0.277; longer sounds: *M* = 0.453; *SD* = 0.229), *F*_(1,9)_ = 0.127, *p* > 0.05, as Figure 6 shows. Although *post-hoc* analyses do not show any significant differences between subgroups, both subgroups differ significantly from controls in both conditions (all *p_s_* < 0.05, ŋ^2^_*p*_ ranging between 0.189 and 0.401). Also in this case, two outliers may be identified in the subgroup of children with surface DD in the processing of long sounds (see Figure 3). Again though, when repeating the main ANOVA without these participants, the interaction Type of Dyslexia x Stimulus Length keeps its significance, *F*_(1, 18)_ = 5.69, *p* < 0.05; ŋ^2^_*p*_ = 0.240.

### Correlations between measures of RAP and reading/reading-related variables

Calculation of correlations was performed for the two groups of dyslexic and normally reading children separately, in order to avoid spurious effects of reading ability (see also Rosen, 2003). To reduce the number of correlations to be computed, compound scores were considered for the RAP variables, including: average of all Rapid-TOJs; average of all Memory-TOJs; average of all TOJs (irrespective of sequence length), average of all TOJs subdivided for length (75 and 250). Considering the results of the previous analyses, two new variables were purposely computed, expressing the difference between accuracy scores with 250 and with 75 ms tones (i.e., the advantage for processing long tones) in all the TOJ tasks (Long-short tones) and the difference between long and short ISIs (Long-short ISIs)^2^. Pattern discrimination scores were also included in the analysis. A first interesting result is the absence of correlations in the control group between RAP variables and age, whereas strong correlations emerged in the group with dyslexia. Since no correlation was found in the DD group between RAP variables and IQ, nor did any correlations emerge between age or IQ and reading scores expressed as z-scores (all *r_s_* < 0.3), Pearson's bivariate correlations were computed including z-scores. The “Long-short tones” difference variable showed the strongest correlations with reading variables (but no correlations with age and IQ), namely with Text and Nonword reading accuracy (*r* = 0.402 and 0.399 respectively, *p* < 0.05) and with overall Nonword reading ability (average of speed and accuracy z-scores) (*r* = 0.554, *p* < 0.005). This variable also showed a correlation with Phoneme deletion (raw score, *r* = −0.438, *p* < 0.05), which was confirmed also when partialling out the effect of Age (*r* = −0.413, *p* < 0.05). Phoneme deletion, in turn, correlated with Pattern discrimination scores, albeit at a very moderate level (*r* = −0.380, *p* = 0.07). Significant correlations emerged also between phonemic blending and word reading speed and accuracy (*r* = 0.451, *p* < 0.05 and *r* = 0.507, *p* < 0.01, respectively) and between phoneme deletion and nonword reading speed and accuracy (*r* = 0.445, *p* < 0.05 and *r* = 0.433, *p* < 0.01, respectively). Correlations with the ISI-related variable (Long-short ISIs) did not reach significance, but it is noteworthy that almost all correlations with reading variables are negative ones, i.e., contrary to what happens with tone length, high sensitivity to differences in ISI predicts lower reading performances. A moderate correlation between the two differential variables was found only for control children (*r* = 0.446, *p* = 0.048).

Multiple linear regressions were additionally performed to further explore the relationship between RAP, reading and reading related measures. A regression analysis (backward method) based on the results of the correlation analysis allowed to predict Nonword reading ability (average of speed and accuracy z-scores) through Phoneme deletion, total Pattern discrimination and the “Long-short tones” difference (entered together with Age and Phonemic blending, which showed no effect on the depending variable). The model was highly significant, *F*_(3, 22)_ = 5.583, *p* = 0.006 and explained 42% of the variance (30% was explained by the “Long-short tones” difference, 12% by Phoneme deletion, and 1% by Pattern discrimination scores). The best predictive model (*F* = 3.506, *p* = 0.047, *R*^2^ = 0.234) for Word reading scores included phonemic blending and the difference between short and long ISIs (both with negative coefficient), that explained, respectively, 14.5 and 8.9%, of the variance. A general reading score expressing the average of word, nonword and text reading, including speed and accuracy, was best predicted (*F* = 5.346, *p* = 0.012, *R*^2^ = 0.317) by both differential scores concerning ISIs (15.8%) and stimulus length (15.9%). Phonemic blending was nonsignificant and added less than 4% to the variance. On the other hand, Phoneme deletion scores could be predicted by “Long-short tones” difference (accounting for 19% of variance) and Pattern discrimination scores (accounting for 13% of variance) - whereas Age had no effect on the model—*F*_(2, 23)_ = 5.195, *p* = 0.014, *R*^2^ = 0.31. No predictive model was found for Phonemic blending scores.

## Discussion

The aim of the present study was to investigate Rapid Auditory Processing (RAP) abilities in Italian children (reading a regular orthography) with DD, subdivided according to age, presence/absence of a previous language delay, and subtype of dyslexia, following the hypothesis that differences in performance among subgroups could explain part of the heterogeneity of results described in the literature concerning RAP.

First of all, the presence of a general auditory processing deficit concerning non-verbal stimuli in Italian children with DD was confirmed, extending the finding obtained in a smaller sample of Italian children with DD (Cantiani et al., 2010), and replicating recent findings concerning other languages with consistent orthographies (Georgiou et al., 2010; Landerl and Willburger, 2010). Children with DD performed worse than their matched controls both in reproducing the order of pairs and sequences of tones, and in judging the equality of 4-tone rhythms. The task that seems to better discriminate between control children and children with DD is the Pattern Discrimination task. In order to exclude that the reading difficulties themselves could be the cause of a suboptimal development of these skills (see Rosen, 2003; Ziegler et al., 2009), a further comparison with a Reading Level (based on Text reading speed) matched group was performed, confirming the presence of a significant difference between children with DD (*n* = 9) and (younger) control children (*n* = 9), *F*_(1, 17)_ = 5.40, *p* < 0.05 [2-tailed]; ŋ^2^_*p*_ = 0.268 (with IQ as covariate).

Specifically concerning the TOJ-Memory task, all participants showed a drop in performance with sequences of 5 sounds compared to sequences of 4 sounds, but children with DD performed worse with the longest sequences, compared to controls. A similar result was obtained by Heiervang et al. (2002), and might point to the role of auditory short-term memory. The influence of short-term memory on reading acquisition is largely supported (Ackerman et al., 1990; Kibby et al., 2004). Moreover, some authors consider short-term memory, and in particular working memory, as a crucial factor influencing performance in RAP tasks (Banai and Ahissar, 2004), also when only two sounds are presented.

*Age* plays a relevant role in the Rapid-TOJ task (see Figure 1). Its effects can be seen in control children only for the most difficult condition, i.e., with the shortest ISIs. By contrast, in children with DD performance improves with age in both conditions, but particularly so in the easier one (longer ISIs) where children with DD finally reach the level of control children. Similarly, in the TOJ-Memory task (see Figure 2) control children show improvements with age in the most difficult conditions (with 4 and 5-tones), but not in the easiest condition (2-tones), probably due to a ceiling effect. Conversely, in children with DD age affects performance in the 2 and 4-tone conditions, but not in the most difficult one (5 tones), which shows a relative floor effect. These results, similar to those found by Hautus et al. (2003) and Tallal (2000), may suggest a relative compensation of RAP deficits in children with DD, with difficulties appearing only when the complexity of the task increases, either through short-term memory load or through faster presentation rates. This may reflect an anomalous or slowed development of short-term memory functions in children with DD. For instance, Nicolson et al. (1992) found in 15-year-old children with DD only a lack of fluency in articulation and slight deficits in memory span, while deficits in phonological processing were no longer detectable.

The results obtained by subdividing children with DD on the basis of the *presence/absence of a language delay* are partly in line with previous findings such as those reported by Tallal and Stark (1982), Heath et al. (1999), and Joanisse et al. (2000), who found auditory processing deficits only in reading-impaired children with a concomitant oral language delay. Indeed, in our sample children with DD and a previous language delay did obtain the lowest scores in RAP measures. However, they showed a pattern of performance similar to that of normally reading children, yielding lower accuracy scores with shorter stimuli. On the other hand, children with DD-only had a more mildly impaired, but more anomalous performance pattern, not showing the advantage for longer stimuli found in the other subgroups. A possible explanation calls into play the effect of language impairment on the use of cognitive strategies during RAP tasks. Indeed, children with DD and a previous language impairment are often characterized by problems with lexical access (Bishop et al., 2009; Chilosi et al., 2009). Following Bretherton and Holmes (2003), performance on RAP tasks may be facilitated by the use of verbal labels to characterize and more easily distinguish or recognize the different sequences, but children with impaired lexical access may have difficulties in establishing and retrieving verbal labels for the tone sequences quickly and accurately. By contrast, dyslexic-only children seem to be characterized by a phonemic awareness deficit—indeed, their performance in phoneme deletion is almost significantly worse than that of children with a previous language impairment (see Table 2). This hypothesis is in line with the findings in English-speaking children with DD with and without oral language impairments, showing that the latter are characterized by more severe phonological deficits and the former by impairment in broader language abilities (Bishop and Snowling, 2004). Nonetheless, the greater impairments in phonemic awareness and verbal memory found in DD-only children does not produce greater impairments in RAP tasks (the hypothesis that RAP deficits are simply a consequence of phonemic awareness deficits also contrasts with data from longitudinal studies such as Benasich and Tallal, 2002; Leppänen et al., 2010). This suggests that other deficits, not related to phonemic awareness and to verbal memory, and also not expressing an effect of worse reading skills (DD+LI children have better reading scores than DD-noLI) must constitute the basis for the significantly lower performance on RAP tasks. Such abilities might have to do with lexical skills, which are a characteristic of SLI children, with and without dyslexia, as shown in many studies (e.g., Chilosi et al., 2009; Nation, 2014).

The present results from children subdivided according to presence/absence of a previous language delay need further explanation. First of all, the language-impaired subgroup does not show lower performances in phonemic awareness and reading scores as usually described in the literature concerning opaque languages such as English (Catts et al., 2005; Ramus et al., 2013). Instead, these children show similar or even better performances with respect to the subgroup without previous language delay. This finding is consistent with other studies on Italian children (Brizzolara et al., 2006; Scuccimarra et al., 2008; Chilosi et al., 2009) where language-impaired children with DD showed no clear disadvantage in reading measures compared to dyslexic-only children, and it may suggest a reduced impact of linguistic deficits on learning regular orthographies. As to the relationship with RAP, it may be hypothesized that a milder but more pervasive deficit as that observed in dyslexic-only children has a greater impact on reading (possibly—as shown by correlation scores—through its effects on phonemic awareness) than a more severe but more “normally modulated” deficit as is observed in children with DD and a previous language delay. Also the inclusion of children for whom a language disorder was present in the past but was then resolved or compensated may have led to unexpected results. Indeed, the presence of early delays in language development that are compensated or resolved before school age is a common report for many children with dyslexia (e.g., Scarborough and Dobrich, 1990; Stothard et al., 1998), and it may well be associated to less severe reading impairment as compared to children with persistent (and probably more pervasive) language disorders. Additionally, published studies on RAP deficits in children with language impairment mostly fail to distinguish children with/without a concomitant reading disorder (e.g., Tallal and Piercy, 1973a,b), and the same is true for studies investigating dyslexic children (e.g., Marshall et al., 2001; Bretherton and Holmes, 2003, etc.), without extending the analysis to (previous and/or concomitant) language abilities—so that previous characterizations of the various subgroups may have been confounded.

Results from the subdivision according to *type of dyslexia* partially support Tallal's findings of a correlation between tone processing and non-word reading (Tallal, 1980). However, we did not find that RAP impairment was specific of children with phonological DD, as was reported by Cestnick (2001). In our sample, the main difference between children with phonological and surface DD relates to sound length (processing longer vs. shorter sounds). In fact, only children with surface DD (similarly to children with DD and previous language delay, and to a certain extent to controls) improved their performance with longer stimuli, showing a more severe difficulty with short sounds. By contrast, children with phonological DD performed similarly in the two conditions. The lack of advantage in recognizing words—which was the criterion for defining these children as “surface” dyslexic—may thus be related to less efficient lexical access, similar to what was suggested for dyslexic children with previous language impairment. In both cases, generally reduced performance on RAP tasks with an otherwise “normal” performance pattern may result from impaired use of cognitive, lexical strategies to facilitate the task. What remains to be explained is the anomalous performance pattern in so-called “phonological dyslexic” children, who have more difficulties in reading nonwords.

Indeed, the interaction found in the present sample between type of phonemic awareness task and type of dyslexia (see the paragraph on the characterization of the subgroups) suggests that the equation between impaired nonword reading and low phonemic awareness skills is a too simplistic one. The direct link between RAP scores and nonword reading (correlations and regression models), and the indirect correlations emerging between nonword reading and phoneme deletion, and between phoneme deletion and pattern discrimination point to a specific bridge between RAP (the advantage in processing long over short tones and rhythmic pattern analysis), the ability to analyze and manipulate (but not to blend) phonemic strings, and nonword reading ability, the latter ability being relatively preserved in surface dyslexic children. In spite of the label of Phonological dyslexia, thus, poor nonword reading does not imply lower performance on phonemic awareness tasks [overall performance on phonemic awareness tasks in this group does not differ at all from that of surface dyslexic children, *p* > 0.99; see (Ziegler et al., 2008) and (Sprenger-Charolles et al., 2000) for similar findings] but rather a *different* type of impairment on phonemic tasks. The role of phoneme deletion as opposed to phonemic blending and its relationship with nonword reading is an interesting one: the ability to analyze and manipulate single phonemes producing nonwords from words (the result of the deletion process) is more relevant to the possibility to handle non-lexical phonemic sequences such as nonwords. Phoneme blending, by contrast, appears more strictly related to the ability to recognize strings of sounds as meaningful units, namely as lexical entries such as words. The importance of phoneme deletion in explaining nonword reading had already been highlighted by (e.g., Pasquini et al., 2007). Nonetheless, one could have expected phonemic blending to predict nonword reading as well, being one of its constituent processes: the absence of such effect calls into play the twofold nature of nonword reading as a phonological and visual task. Indeed, strong relationships have been demonstrated between visual-spatial attentional deficits and nonword reading in Italian children with DD (Facoetti et al., 2006, 2010; Franceschini et al., 2012) suggesting that reading ordered strings of letters not only requires phonological ability, but also visual-spatial attentional skills. Furthermore, attentional deficits in the visual and auditory modality seem to follow similar pathways (Facoetti et al., 2003, 2010). It may be thus be hypothesized that the children showing more problems in the auditory modality correspond to DD+LI (language-impaired, here showing the lowest scores on RAP tasks), while children with phonological dyslexia (who show both the lowest scores and the most anomalous pattern on RAP tasks) may suffer from problems involving both the auditory and the visual modality (thus possibly more pervasive and with a more severe expression).

As a conclusion, the present findings are best interpreted within a multifactor model of dyslexia (see Pennington, 2006; Boets et al., 2007), which takes into account the effects of variables related to developmental trends and specific neuropsychological profiles. Further, it may suggest that other variables, especially related to verbal memory, lexical processing and attentional functions play a role in modulating the relationship between auditory temporal processing of nonverbal stimuli, phonemic skills and reading abilities. Since lexical access and attention have not been directly measured in the present study, their involvement follows from more speculative reasons and deserves further investigation. The lack of advantage for processing longer stimuli seems to be a very crucial issue, possibly indicating a more pervasive deficit in RAP (whatever the exact nature of this process and the mechanisms implied), which can produce detrimental effects on reading—especially when interacting with concomitant impairments at the visual-attentional level. Last but not least, the correlations between RAP variables, phonemic awareness skills and reading support the idea that processing auditory stimuli is not simply an associated problem, but concurs in determining both the quality and quantity of the reading deficit, in strict interplay with other variables. These findings thus do not support simplified versions of the RAP deficit, just focusing on deficits in processing short and rapid sounds, and rather depict a far more complex model of the interrelations between the different variables. Very crucial is the finding that RAP variables, not expressing absolute performance levels but rather differential scores describing the level of sensitivity to changes in tone length or ISI, are the best predictors of reading abilities in their various aspects, and better predictors than phonemic awareness and verbal memory skills. Even further, such differential variables contribute to the prediction of specific forms of phonemic awareness itself. Interestingly, while higher sensitivity to changes in tone length is associated to better reading performance, higher sensitivity to ISI changes are associated with worse reading performance. A closer inspection of correlation patterns in DD children suggests, in fact, that increased ISI-related differences depend on better performance with the longest ISIs (*r* = 0.630, *p* = 0.001, i.e., they express greater ability to take advantage from increases in ISI) whereas increased differences with respect to tone length depend on lower performances with short tones (*r* = −0.371, *p* = 0.062, i.e., they express more severe impairments).

The limited number of participants in each of the various subgroups calls for caution in generalizing its results. Further limitations of the study are the absence of concomitant language and attention measures, and the use of previous clinical reports (in a few cases, parents' reports) to identify children with comorbid language impairments. Nonetheless, the relatively homogeneous profiles within each group and the replication of some of the results in reading-level matched comparisons support their validity, and may offer stimulating hints as to the range and type of variables that need to be taken into account when investigating sensory processing in developmental disorders and their relationship with reading skills. The crucial role played by RAP variables (related to length of the stimuli, ISI, and to sensitivity to their modulations) in predicting specific aspects of reading performance (with differential effects on word and nonword reading) suggests that addressing such skills in intervention programs and choosing specific RAP targets according to the specific reading patterns may be an effective and innovative rehabilitation strategy. Furthermore, the present results suggest that linguistic variables (possibly at the lexical level) different from memory and phonemic awareness influence RAP and reading performance in children with comorbid DD and (even if compensated) LI. Further research seems to be necessary for the identification and characterization of such variables, that could shed better light on the complex relationships between low- and high-level processing of language.

### Conflict of interest statement

The authors declare that the research was conducted in the absence of any commercial or financial relationships that could be construed as a potential conflict of interest.

## Footnotes

^1^Although it is sometimes argued that mistakes are very rare in transparent orthographies (e.g., Landerl et al., 1997), it was clearly shown that children with DD do make mistakes (though less numerous than is described for opaque languages), even in a language with a very consistent orthography as Italian (Brizzolara et al., 2006; Menghini et al., 2010).

^2^This variable was computed within each subject by regressing ln(ISI) onto performance and using predicted performance for the maximum and the minimum ISI. Then, the score related to the minimum ISI was subtracted from the score related to the maximum ISI.
